# Clinical and OCT Predictors of Refractory Vogt–Koyanagi–Harada Disease

**DOI:** 10.1016/j.xops.2026.101123

**Published:** 2026-02-18

**Authors:** Yang Cheul Cho, Yuki Mizuki, Jutaro Nakamura, Akira Meguro, Shun Kanasashi, Takuto Sakono, Tatsukata Kawagoe, Shigeaki Ohno, Nobuhisa Mizuki

**Affiliations:** 1 Department of Ophthalmology and Visual Science, Yokohama City University Graduate School of Medicine, Yokohama, Japan; 2 Department of Ophthalmology, Yokohama Minami Kyousai Hospital, Yokohama, Japan; 3 Department of Ophthalmology, Dokkyo Medical University, Mibu, Japan; 4 Department of Ophthalmology, Faculty of Medicine and Graduate School of Medicine, Hokkaido University, Sapporo, Japan

**Keywords:** Vogt-Koyanagi-Harada disease, Uveitis, Optical coherence tomography, Bacillary layer detachment, Refractory disease

## Abstract

**Objective:**

To identify baseline clinical and OCT predictors of refractoriness in Vogt–Koyanagi–Harada (VKH) disease.

**Design:**

A retrospective cohort study.

**Participants:**

Two hundred thirty-seven patients (472 eyes) with VKH disease seen at Yokohama City University Hospital between November 2009 and March 2024.

**Methods:**

Patients were diagnosed according to the revised international diagnostic criteria and all received systemic corticosteroid pulse therapy. Baseline demographic, clinical, and OCT findings were collected, and logistic regression analyses were performed to evaluate associations with refractory disease, defined as ≥2 recurrences or the need for additional immunosuppressive therapy. The study was approved by the institutional review board and conducted in accordance with the Declaration of Helsinki.

**Main Outcome Measures:**

Associations between baseline clinical and OCT findings, including visual acuity (VA), central retinal thickness (CRT), bacillary layer detachment (BALAD), and interval from onset to pulse therapy, and the development of refractory VKH.

**Results:**

Twenty-six patients (11.0%; 52 eyes) developed refractory VKH, whereas 211 patients (89.0%; 420 eyes) were nonrefractory. In the primary multivariable analysis, worse baseline VA (odds ratio [OR], 1.53; 95% confidence interval [CI], 1.19–1.96; *P* < 0.001), greater CRT (OR, 1.66; 95% CI, 1.19–2.32; *P* = 0.003), and a longer interval to initiation of pulse therapy (OR, 1.27; 95% CI, 1.02–1.57; *P* = 0.031) were significantly associated with refractoriness, whereas female sex was associated with lower odds of refractory VKH (OR, 0.50; 95% CI, 0.26–0.96; *P* = 0.039) and age at onset was not. Bacillary layer detachment, although frequent in the acute stage, was not associated with refractoriness. In a sensitivity analysis additionally adjusting for the number of pulse therapy courses, only baseline VA and the number of pulse courses remained significantly associated with refractoriness.

**Conclusions:**

Poor baseline VA was the most consistent predictor of refractory VKH. Greater CRT, delayed initiation of pulse therapy, and female sex showed associations in the primary model but should be regarded as candidate predictors that require confirmation, whereas BALAD was not associated with refractoriness.

**Financial Disclosure(s):**

The authors have no proprietary or commercial interest in any materials discussed in this article.

Vogt–Koyanagi–Harada (VKH) disease is a systemic autoimmune disorder caused by an autoimmune response against melanocytes, leading to inflammation in the central nervous system, eyes, skin, and ears. Visual outcomes depend on the severity of ocular involvement and the adequacy of treatment. The acute stage is typically characterized by bilateral granulomatous panuveitis with exudative (serous) retinal detachment.^1^^,^^2^ Clinical courses of VKH are heterogeneous. Urzua et al^3^ described 2 distinctive courses of VKH: initial-onset acute and chronic recurrent disease. The chronic recurrent form is generally more refractory to treatment and associated with a higher rate of complications. Systemic corticosteroid therapy remains the cornerstone of management, and accumulating evidence suggests that intensive treatment in the early phase may reduce the risk of chronic evolution, supporting the concept of a “therapeutic window.”^4^, ^5^, ^6^

OCT has enabled detailed characterization of acute VKH and may provide prognostic information on refractoriness. Multiple exudative retinal detachments (multi-ERDs) and choroidal folds are common,^7^^,^^8^ and bacillary layer detachment (BALAD) has recently been recognized as a frequent finding.^9^ Some studies have suggested that BALAD indicates more severe disease and may predict recurrence or long-term complications,^10^^,^^11^ whereas others found no prognostic value.^12^ In addition, central retinal thickness (CRT) may reflect the degree of exudation but has not been consistently associated with refractoriness.^13^ However, findings have not been consistent across studies, and variable definitions of treatment resistance further limit comparability.

To date, reliable baseline predictors of refractoriness in VKH remain uncertain. This study aimed to identify clinical and OCT predictors of refractory VKH in a consecutive cohort from a tertiary referral center.

## Methods

### Study Design and Participants

This retrospective study was conducted at Yokohama City University Hospital between November 2009 and March 2024. Patients were eligible if they were diagnosed with VKH disease according to the Revised Diagnostic Criteria proposed by the International Committee in 2001.^14^ Exclusion criteria were as follows: patients without available pretreatment OCT data; patients younger than 18 years; those who did not receive systemic corticosteroid pulse therapy; and those who initiated pulse therapy with a reduced dosage regimen.

The study protocol was approved by the Institutional Review Board of Yokohama City University (approval number: F250700001) and was conducted in accordance with the tenets of the Declaration of Helsinki. Informed consent was obtained using an opt-out approach on the hospital website.

### Clinical Data Collection

Baseline demographic and clinical information, including age at onset, sex, and use of systemic immunosuppressive therapy, were collected for each patient. Disease onset was defined as the day when prodromal symptoms such as headache, tinnitus, or meningismus first appeared, if present, or otherwise the day when ocular symptoms first appeared. Ocular findings at presentation were assessed in 472 eyes and included the presence of multi-ERD, choroidal folds, BALAD, subretinal fibrin, anterior segment inflammation, choroidal detachment, optic disc hyperemia, sunset glow fundus, posterior synechiae, keratic precipitates, choroidal neovascularization, and vitreous opacity.

OCT was performed using the Heidelberg Spectralis system (Heidelberg Engineering). Central retinal thickness was manually measured with the built-in caliper function from the internal limiting membrane to the retinal pigment epithelium, including subretinal fluid if present. Best-corrected visual acuity (VA) was recorded and converted to the logarithm of the minimum angle of resolution. OCT findings were independently evaluated by 3 ophthalmologists (Y.C.C., Y.M., and J.N.). Discrepancies in interpretation were resolved by consensus discussion. Ellipsoid zone, interdigitation zone, and choroidal thickness could not be reliably assessed in the acute phase and were not included.

Refractory VKH was defined as either ≥2 recurrences after the initial onset or the requirement for additional immunosuppressive agents such as cyclosporine or adalimumab. Recurrence was defined as inflammation uncontrolled by escalation of topical corticosteroid therapy alone, requiring systemic corticosteroid pulse therapy, increased oral corticosteroid dosage, or the initiation of immunosuppressive agents. The inflammation could involve anterior segment activity (an increase in anterior chamber cells with or without flare), posterior segment activity (serous retinal detachment, choroidal folds, or subretinal dye pooling on fluorescein angiography), or both.

### Treatment Variables

Initial corticosteroid pulse therapy was administered in all patients as intravenous methylprednisolone 500 mg/day for 3 days (1 course). Additional pulse courses were not predetermined by baseline findings but were decided according to early treatment response, particularly the persistence of subretinal dye pooling on fluorescein angiography. Additional courses were usually given at approximately 1 week after the preceding course. Following pulse therapy, all patients received oral prednisolone at an initial dose of 1 mg/kg/day, with tapering determined by the clinical response. The interval from disease onset to initiation of pulse therapy was recorded in days. In most patients, this interval reflected delayed presentation to an ophthalmologist or delayed referral to our tertiary center, as systemic corticosteroid pulse therapy was generally initiated promptly once VKH was diagnosed.

Systemic immunosuppressive agents such as cyclosporine and adalimumab were introduced as step-up therapy in patients with recurrent or persistent inflammation despite high-dose systemic corticosteroids or difficulty tapering oral corticosteroids and were not used as upfront early combination therapy at initial presentation in this cohort.

### Statistical Analysis

The primary outcome was refractory VKH, as defined in the Clinical Data Collection section. Baseline characteristics were summarized as number (percentage) for categorical variables and median (interquartile range) for continuous variables. Group differences between refractory and nonrefractory patients were assessed using Fisher exact test for categorical variables and the Mann–Whitney *U* test for continuous variables. Predictors of refractoriness were then assessed using logistic regression. Continuous variables were standardized per 1 standard deviation, and binary variables were analyzed as present versus absent (or female vs. male). Odds ratios (ORs) with 95% confidence intervals (CIs) were calculated, and *P* values from the univariate analyses were adjusted for multiple testing using the Benjamini–Hochberg false discovery rate (FDR) method. Sunset glow fundus and choroidal neovascularization were considered sequelae of refractory disease and excluded from regression.

Variables for multivariable logistic regression were selected with reference to FDR-adjusted univariate results. Age at onset was included as a demographic factor, while the number of initial pulse therapy courses was excluded from the primary model because it reflects early treatment response and treatment intensity during the acute phase rather than a baseline prognostic factor. Sensitivity analyses were conducted by including BALAD and by including pulse courses; both models were estimated with Firth penalized logistic regression to address sparse data bias. Analyses were performed in R (version 4.5.1), with 2-sided *P* < 0.05 considered significant.

## Results

### Patient Characteristics

**Figure 1:**
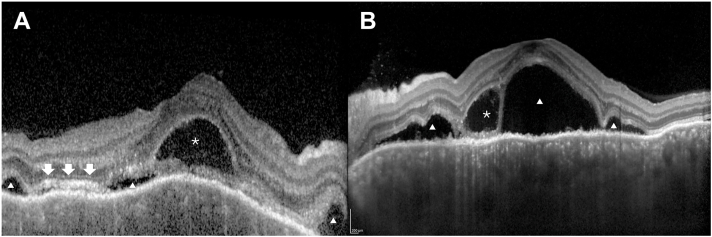
Representative OCT findings in acute Vogt–Koyanagi–Harada disease. **A,** OCT image with BALAD (asterisk), exudative retinal detachment (ERD, triangle), and subretinal fibrin (arrow). **B,** OCT image demonstrating BALAD (asterisk) and ERD (triangle). BALAD = bacillary layer detachment; ERD = exudative retinal detachment.

A total of 237 patients with VKH were included; 26 (11.0%) developed refractory disease, whereas 211 (89.0%) were nonrefractory (Table 1). Of these 26 refractory patients, 16 (61.5%) received systemic immunosuppressive therapy as step-up treatment. Refractory patients were more often male (69.2% vs. 44.1%, *P* = 0.001), whereas age at onset was similar between groups (median 58 vs. 52 years, *P* = 0.245). They more frequently exhibited sunset glow fundus (53.8% vs. 11.2%, *P* < 0.001) and had multi-ERD more frequently (88.5% vs. 74.8%, *P* = 0.037). The median CRT was greater in refractory eyes (median 1036 vs. 817 μm, *P* < 0.001). Refractory patients also received more initial pulse therapy courses (median 2 vs. 1, *P* < 0.001) and had a longer interval to initiation (median 22 vs. 16 days, *P* = 0.007). During follow-up, they experienced more relapses (median 2 vs. 0, *P* < 0.001) and had longer follow-up durations (median 4.0 vs. 1.0 years, *P* < 0.001). Representative OCT images are shown in Figure 1.

**Table 1:** Baseline Patient, Ocular, and Treatment Characteristics

Patient Characteristics	All (n = 237)	Refractory VKH (n = 26)	Nonrefractory VKH (n = 211)	*P* Value
Sex				
Male	111 (46.8%)	18 (69.2%)	93 (44.1%)	
Female	126 (53.2%)	8 (30.8%)	118 (55.9%)	0.001
Age at onset	52 (41–65)	58 (47–64)	52 (40–65)	0.245
Immunosuppressive therapy	16 (6.8%)	16 (61.5%)	0 (0.0%)	<0.001

Eye characteristics	All (n = 472)	Refractory VKH (n = 52)	Nonrefractory VKH (n = 420)	*P* Value
Right eyes	237 (50.2%)	26 (50.0%)	209 (49.8%)	
Left eyes	235 (49.8%)	26 (50.0%)	211 (50.2%)	1.000
Lens status				
Phakia	441 (93.4%)	42 (80.8%)	399 (95.0%)	
Pseudophakia	31 (6.6%)	10 (19.2%)	21 (5.0%)	<0.001
Multi-ERD	360 (76.3%)	46 (88.5%)	314 (74.8%)	0.037
Choroidal fold	360 (76.3%)	45 (86.5%)	315 (75.0%)	0.083
BALAD	268 (56.8%)	31 (59.6%)	237 (56.4%)	0.767
Subretinal fibrin	39 (8.3%)	6 (11.5%)	33 (7.9%)	0.419
Anterior segment inflammation	269 (57.0%)	34 (65.4%)	235 (56.0%)	0.235
Choroidal detachment	19 (4.1%)	4 (7.7%)	15 (3.6%)	0.146
Optic disc hyperemia	346 (73.3%)	42 (80.8%)	304 (72.4%)	0.245
Sunset glow fundus	75 (15.9%)	28 (53.8%)	47 (11.2%)	<0.001
Posterior synechiae	30 (6.4%)	4 (7.7%)	26 (6.2%)	0.560
KPs	32 (6.8%)	2 (3.8%)	30 (7.1%)	0.560
CNV	10 (2.1%)	4 (7.7%)	6 (1.4%)	0.017
Vitreous opacity	35 (7.4%)	8 (15.4%)	27 (6.4%)	0.042
CRT (μm)	840 (617–1068)	1036 (801–1226)	817 (600–1024)	<0.001
Baseline VA (logMAR)	0.10 (0.00–0.40)	0.40 (0.10–0.77)	0.10 (–0.08–0.40)	<0.001
Final VA (logMAR)	–0.08 (–0.08–0.05)	0.05 (–0.08–0.30)	–0.08 (–0.08–0.00)	<0.001
Change in VA (logMAR)	–0.12 (–0.38–0.00)	–0.24 (–0.66–0.00)	–0.10 (–0.38–0.00)	0.034
Number of relapses	0 (0–0), range 0–7	2 (1–2), range 0–7	0 (0–0), range 0–1	<0.001

Treatment characteristics	All (n = 237)	Refractory VKH (n = 26)	Nonrefractory VKH (n = 211)	*P* Value
Initial pulse therapy (courses)	1 (1–2), range 1–12	2 (1–3), range 1–12	1 (1–1), range 1–3	<0.001
Pulse daily dose (g/day)	0.50 (all patients)	0.50 (all patients)	0.50 (all patients)	
Days to pulse therapy	17 (12–25), range 4–352	22 (13–37), range 5–272	16 (11.5–24), range 4–352	0.007
Follow-up period (years)	1.0 (1.0–2.0), range 0.6–14	4.0 (3.0–6.5), range 1.0–14	1.0 (1.0–1.5), range 0.8–13	<0.001

BALAD = bacillary layer detachment; CNV = choroidal neovascularization; CRT = central retinal thickness; KPs = keratic precipitates; logMAR = logarithm of the minimum angle of resolution; multi-ERD = multiple exudative retinal detachment; VA = visual acuity; VKH = Vogt–Koyanagi–Harada disease.

Values are presented as n (%) or median (interquartile range). *P* values were calculated using Fisher exact test for categorical variables and the Mann–Whitney *U* test for continuous variables. Pulse daily dose was identical (0.50 g/day) in all patients; therefore, no *P* value was calculated.

### Univariate Analysis

**Figure 2:**
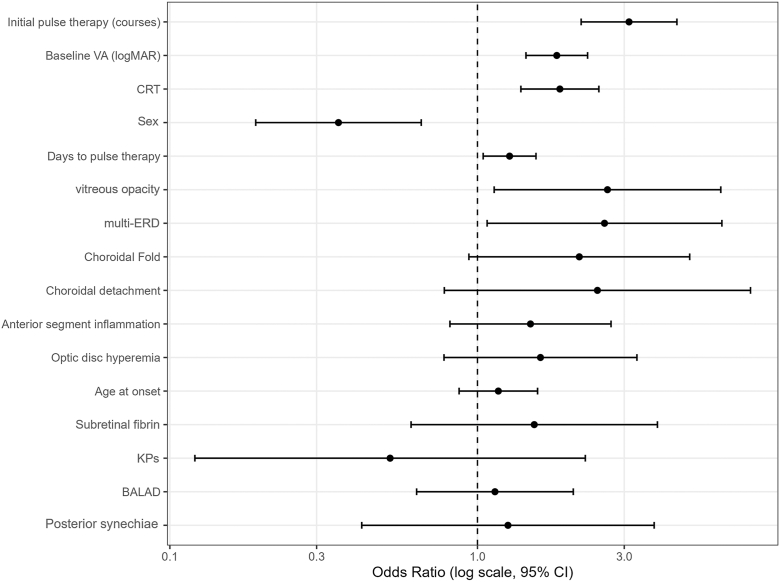
Univariate logistic regression analysis for refractory VKH disease. Forest plot of univariate logistic regression analyses for refractory VKH disease. Odds ratios and 95% CIs are shown (per 1 SD for continuous variables). Detailed numerical results are provided in Table 2. BALAD = bacillary layer detachment; CI = confidence interval; CRT = central retinal thickness; KPs = keratic precipitates; logMAR = logarithm of the minimum angle of resolution; multi-ERD = multiple exudative retinal detachment; SD = standard deviation; VA = visual acuity; VKH = Vogt–Koyanagi–Harada.

In univariate analyses, several baseline factors were significantly associated with refractory VKH. A greater number of initial pulse therapy courses (OR, 3.11; 95% CI, 2.17–4.45; *P* < 0.001), worse baseline VA (OR, 1.81; 95% CI, 1.44–2.28; *P* < 0.001), increased CRT (OR, 1.85; 95% CI, 1.38–2.48; *P* < 0.001), and female sex (OR, 0.35; 95% CI, 0.19–0.66; *P* < 0.001) remained significant after FDR adjustment. Days to pulse therapy, vitreous opacity, and multi-ERD showed nominal associations (*P* < 0.05) but were not significant after correction. Other ocular findings, including BALAD and subretinal fibrin, showed no association with refractoriness (Fig 2, Table 2).

**Table 2:** Univariate Logistic Regression Analyses for Refractory Vogt–Koyanagi–Harada (VKH) Disease

Variable	Type	Scale	OR (95% CI)	*P* Value	Q Value (FDR)
Initial pulse therapy (courses)	continuous	per 1 SD (0.94)	3.11 (2.17–4.45)	<0.001	<0.001
Baseline VA (logMAR)	continuous	per 1 SD (0.41)	1.81 (1.44–2.28)	<0.001	<0.001
CRT	continuous	per 1 SD (352.27)	1.85 (1.38–2.48)	<0.001	<0.001
Sex (female vs. male)	binary	—	0.35 (0.19–0.66)	<0.001	0.004
Days to pulse therapy	continuous	per 1 SD (30.69)	1.27 (1.04–1.55)	0.017	0.055
Vitreous opacity (present vs. absent)	binary	—	2.65 (1.13–6.18)	0.025	0.065
Multi-ERD (present vs. absent)	binary	—	2.59 (1.07–6.23)	0.034	0.078
Choroidal fold (present vs. absent)	binary	—	2.14 (0.94–4.90)	0.071	0.141
Choroidal detachment (present vs. absent)	binary	—	2.45 (0.78–7.72)	0.125	0.222
Anterior segment inflammation (present vs. absent)	binary	—	1.49 (0.81–2.72)	0.197	0.292
Optic disc hyperemia (present vs. absent)	binary	—	1.60 (0.78–3.30)	0.200	0.292
Age at onset	continuous	per 1 SD (15.97)	1.17 (0.87–1.57)	0.297	0.396
Subretinal fibrin (present vs. absent)	binary	—	1.53 (0.61–3.85)	0.366	0.435
KPs (present vs. absent)	binary	—	0.52 (0.12–2.24)	0.380	0.435
BALAD (present vs. absent)	binary	—	1.14 (0.63–2.05)	0.662	0.683
Posterior synechiae (present vs. absent)	binary	—	1.26 (0.42–3.75)	0.683	0.683

BALAD = bacillary layer detachment; CI = confidence interval; CRT = central retinal thickness; FDR = false discovery rate; KPs = keratic precipitates; logMAR = logarithm of the minimum angle of resolution; multi-ERD = multiple exudative retinal detachments; OR = odds ratio; SD = standard deviation; VA = visual acuity.

Analyses were performed using univariate logistic regression. Continuous variables were standardized and expressed per 1 SD. Binary variables were analyzed as present vs. absent (or female vs. male for sex). Odds ratios (ORs) are presented with 95% CIs. *P* values were adjusted for multiple testing using the Benjamini–Hochberg FDR method.

### Multivariable Analysis

**Figure 3:**
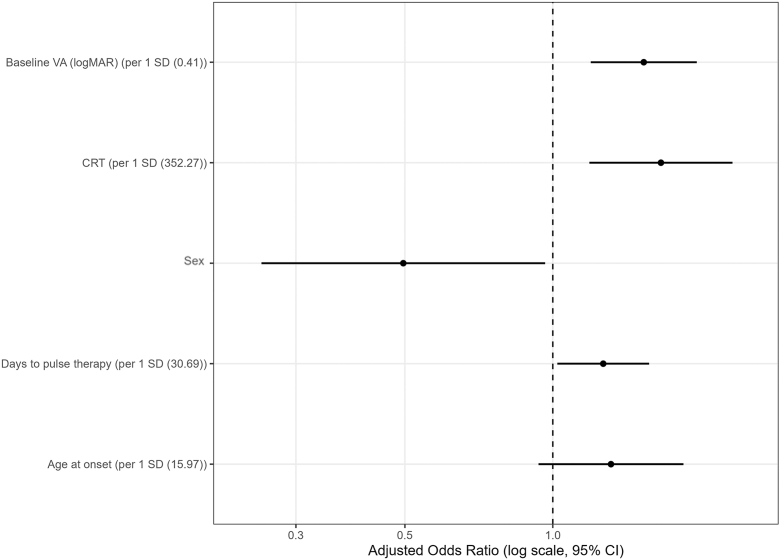
Multivariable logistic regression analysis for refractory VKH disease. Forest plot of multivariable logistic regression analyses. Continuous variables were standardized and expressed per 1 SD. Binary variables were analyzed as present vs. absent (or female vs. male for sex). Odds ratios (ORs) are presented with 95% CIs. CI = confidence interval; CRT = central retinal thickness; logMAR = logarithm of the minimum angle of resolution; SD = standard deviation; VA = visual acuity; VKH = Vogt–Koyanagi–Harada.

Variables for multivariable logistic regression were selected based on univariate results with reference to FDR-adjusted *P* values; days to pulse therapy was included for clinical relevance, whereas the number of initial pulse courses was excluded from the primary model to avoid confounding by treatment intensity. Multivariable analysis showed that worse baseline VA (OR, 1.53; 95% CI, 1.19–1.96; *P* < 0.001), greater CRT (OR, 1.66; 95% CI, 1.19–2.32; *P* = 0.003), and a longer interval to pulse therapy (OR, 1.27; 95% CI, 1.02–1.57; *P* = 0.031) were significantly associated with refractory VKH, whereas female sex was associated with lower odds of refractory VKH (OR, 0.50; 95% CI, 0.26–0.96; *P* = 0.039) and age at onset was not (*P* = 0.116). Results are shown in Figure 3 and Table 3.

**Table 3:** Multivariable Logistic Regression Analysis for Refractory Vogt–Koyanagi–Harada (VKH) Disease

Variable	Scale Label	OR (95% CI)	*P* Value
Baseline VA (logMAR)	per 1 SD (0.41)	1.53 (1.19–1.96)	<0.001
CRT	per 1 SD (352.27)	1.66 (1.19–2.32)	0.003
Sex	female vs. male	0.50 (0.26–0.96)	0.039
Days to pulse therapy	per 1 SD (30.69)	1.27 (1.02–1.57)	0.031
Age at onset	per 1 SD (15.97)	1.31 (0.94–1.84)	0.116

CI = confidence interval; CRT = central retinal thickness; logMAR = logarithm of the minimum angle of resolution; OR = odds ratio; SD = standard deviation; VA = visual acuity.

Analyses were performed using multivariable logistic regression. Continuous variables were standardized and expressed per 1 SD. Binary variables were analyzed as present vs. absent (or female vs. male for sex). Odds ratios are presented with 95% CIs.

### Sensitivity Analysis

Because BALAD has been reported as a marker of more severe disease, we performed a sensitivity analysis including BALAD in the multivariable model using Firth penalized logistic regression. Baseline VA, CRT, days to pulse therapy, and female sex remained significantly associated with refractory VKH, whereas age at onset and BALAD were not significant (Fig S1, Table S1, available at www.ophthalmologyscience.org).

As the number of pulse therapy courses may reflect both baseline disease severity and treatment intensity, this variable was excluded from the main model. In a sensitivity analysis including this factor, baseline VA remained significant, and the number of pulse courses was strongly associated with refractoriness, whereas the associations for CRT, sex, days to pulse therapy, and age at onset were attenuated and were no longer statistically significant (Fig S2, Table S2, available at www.ophthalmologyscience.org).

## Discussion

In our cohort of 237 patients with VKH disease, worse baseline VA was consistently associated with refractory VKH. Greater CRT, a longer interval from disease onset to initiation of pulse therapy, and sex showed significant associations with refractoriness in the primary multivariable model. However, when we additionally adjusted for the number of initial pulse therapy courses in sensitivity analyses, only baseline VA and the number of pulse courses remained significantly associated with refractory disease, whereas the associations for CRT, interval to pulse therapy, and sex were attenuated and no longer statistically significant. These variables should therefore be regarded as potential rather than definitive predictors and interpreted with caution. Bacillary layer detachment, although previously reported as a marker of more severe disease,^11^ showed no significant association with refractoriness in any of our models.

Although our refractory rate (11.0%) appears lower than that reported in prior studies,^13^^,^^15^^,^^16^ direct comparisons are limited by differences in outcome definitions. In our study, recurrence was defined as inflammation requiring escalation beyond topical corticosteroids, and refractoriness was defined as ≥2 such recurrences or the need for additional immunosuppressive agents; this definition may explain the lower refractoriness rate observed in our cohort.

In this cohort, additional pulse courses were determined during the acute phase according to early fluorescein angiography findings, particularly the persistence of subretinal dye pooling within areas of serous retinal detachment, rather than by baseline characteristics. The number of pulse therapy courses therefore represents a marker of early treatment response and treatment intensification during the acute phase rather than a purely baseline prognostic factor, which may partly explain why adjustment for this variable attenuated the associations observed for CRT, treatment delay, and sex.

Consistent with our findings, Feng et al^17^ reported that patients with chronic-recurrent VKH had worse baseline VA than those with acute resolution. However, in their multivariate analysis, baseline VA was no longer significant, with older age and sunset glow fundus identified as predictors. Because sunset glow fundus develops as a consequence of refractory disease, its inclusion may have masked the role of baseline vision. In contrast, our analysis focused only on baseline predictors and demonstrated that poor initial vision is an important indicator of refractoriness. Similarly, Maruyama et al^15^ found that lower baseline VA was associated with recurrence, supporting the importance of visual function at presentation.

Previous studies have suggested that greater serous retinal detachment extent is associated with poor prognosis,^18^ whereas the association between phenotypic classifications, such as serous retinal detachment or papillitis-type and refractoriness, has been inconsistent.^19^^,^^20^ More recently, Hirota et al^13^ reported in a small cohort that CRT itself was not predictive of recurrence, while retinal pigment epithelium reflectivity might be a potential marker. However, quantification of retinal pigment epithelium reflectivity is not standardized, limiting its clinical utility. In our study, greater CRT was associated with refractoriness in the primary multivariable model, suggesting that this established OCT marker may serve as a practical predictor in a large cohort; however, this association was not robust across analyses and should be interpreted with caution.

Delayed initiation of systemic corticosteroids has been reported to increase the risk of recurrence and complications, whereas early intensive therapy improves outcomes.^5^^,^^6^^,^^16^^,^^21^ Consistent with these observations, a longer interval from onset to treatment initiation was associated with refractory disease in our primary multivariable model, lending support to the concept of a “therapeutic window of opportunity” in the acute stage of VKH^4^ and suggesting that early intervention may be important.

In our cohort, female sex showed a significant protective association with refractory VKH in both univariate and primary multivariable analyses. Previous studies have suggested a better prognosis in females and proposed hormone-mediated immunomodulation as a potential explanation, with estrogen and progesterone exerting protective effects on uveitic activity.^22^ This finding is consistent with a possible role of sex-related immunobiology, but the sex-related association observed in our study should be interpreted with caution.

The clinical significance of BALAD in acute VKH remains controversial. While some studies reported associations with poorer vision, recurrence, or long-term complications,^10^^,^^11^ others found no impact on prognosis.^12^ In addition, a recent report found that the absence of BALAD on macular OCT was associated with treatment-refractory VKH.^16^ In our large cohort, BALAD was not significantly associated with refractoriness, suggesting it reflects acute inflammatory activity rather than a prognostic marker.

Taken together, while our findings provide important insights into predictors of refractory VKH, it should be acknowledged that the interpretation of chronic inflammation and refractoriness varies across studies, and direct comparisons must be made with caution given the differences in study design, patient populations, and outcome definitions. In particular, Gin et al^16^ recently identified greater anterior chamber and vitreous inflammation, the absence of macular BALAD, and delayed initiation of systemic steroid therapy as predictive factors for refractory VKH in initial-onset acute disease, and our results partly support the importance of treatment timing while suggesting that the prognostic value of inflammatory signs and BALAD may be less consistent in a larger, broader cohort.

In conclusion, worse baseline VA emerged as a robust predictor of refractory VKH in our cohort. Greater CRT, delayed initiation of pulse therapy, and female sex (with a protective association) also showed associations with refractoriness in the primary multivariable model; however, these variables did not remain statistically significant in sensitivity analyses and should be regarded as candidate predictors that require confirmation. Bacillary layer detachment was not associated with refractoriness. These results suggest that patients with poor baseline vision, and possibly those with increased CRT or treatment delay, may be at higher risk of developing refractory disease, and careful monitoring and consideration of early intensive management may therefore be warranted in such cases.

This study has several limitations. First, it was retrospective and conducted at a single tertiary center, which may limit generalizability. Second, although a relatively large number of patients were included, the number of refractory cases was small, reducing statistical power. Third, certain OCT features, such as the ellipsoid and interdigitation zones, could not be reliably assessed in the acute phase. Fourth, analyses combined patient-level and eye-level variables, an inherent challenge in bilateral diseases such as VKH. Finally, residual confounding cannot be excluded despite multivariable adjustment, and prospective studies are warranted.
