# Supplementary material for: Clinical and OCT Predictors of Refractory Vogt–Koyanagi–Harada Disease
Source: Ophthalmol Sci. 2026 Feb 18;6(4):101123. doi: 10.1016/j.xops.2026.101123 (PMC13011244; doi:10.1016/j.xops.2026.101123)
Supplement: Table S1 [file mmc1.pdf]

Supplementary Table S1. Sensitivity analysis including bacillary layer detachment (BALAD) using Firth's penalized logistic regression.

Variable	Scale Label	OR (95% CI)	p value
Baseline VA (logMAR)	per 1 SD (0.41)	1.51 (1.19–1.94)	<0.001
CRT	per 1 SD (352.27)	1.73 (1.22–2.46)	0.002
Sex	female vs. male	0.52 (0.26–0.99)	0.047
Days to pulse therapy	per 1 SD (30.69)	1.25 (1.02–1.55)	0.029
Age at onset	per 1 SD (15.97)	1.26 (0.90–1.79)	0.181
BALAD	present vs. absent	0.73 (0.36–1.48)	0.376

Analyses were performed using multivariable logistic regression with Firth's penalization. Continuous variables were standardized and expressed per 1 standard deviation (SD). Binary variables were analyzed as present vs. absent (or female vs. male for sex). Odds ratios (OR) are presented with 95% confidence intervals (CI).

Abbreviations

VA = visual acuity; CRT = central retinal thickness; BALAD = bacillary layer detachment.
