# Supplementary material for: Clinical and OCT Predictors of Refractory Vogt–Koyanagi–Harada Disease
Source: Ophthalmol Sci. 2026 Feb 18;6(4):101123. doi: 10.1016/j.xops.2026.101123 (PMC13011244; doi:10.1016/j.xops.2026.101123)
Supplement: Table S2 [file mmc2.pdf]

Supplementary Table S2. Sensitivity analysis including the number of initial pulse therapy courses using Firth's penalized logistic regression.

Variable	Scale Label	OR (95% CI)	p value
Baseline VA (logMAR)	per 1 SD (0.41)	1.50 (1.17–1.93)	0.002
CRT	per 1 SD (352.27)	1.29 (0.91–1.83)	0.155
Sex	female vs. male	0.56 (0.28–1.11)	0.097
Days to pulse therapy	per 1 SD (30.69)	1.16 (0.94–1.44)	0.154
Age at onset	per 1 SD (15.97)	1.19 (0.84–1.72)	0.324
Initial pulse therapy (courses)	per 1 course	2.46 (1.63–3.71)	<0.001

Analyses were performed using multivariable logistic regression with Firth's penalization. Continuous variables were standardized and expressed per 1 standard deviation (SD). Binary variables were analyzed as present vs. absent (or female vs. male for sex). Odds ratios (OR) are presented with 95% confidence intervals (CI).
The number of initial pulse therapy courses was treated as a discrete variable, and odds ratios were calculated per additional course.

Abbreviations
VA = visual acuity; CRT = central retinal thickness.
