# Supplementary material for: Clinical and OCT Predictors of Refractory Vogt–Koyanagi–Harada Disease
Source: Ophthalmol Sci. 2026 Feb 18;6(4):101123. doi: 10.1016/j.xops.2026.101123 (PMC13011244; doi:10.1016/j.xops.2026.101123)
Supplement: Figure S1 [file mmc3.pdf]

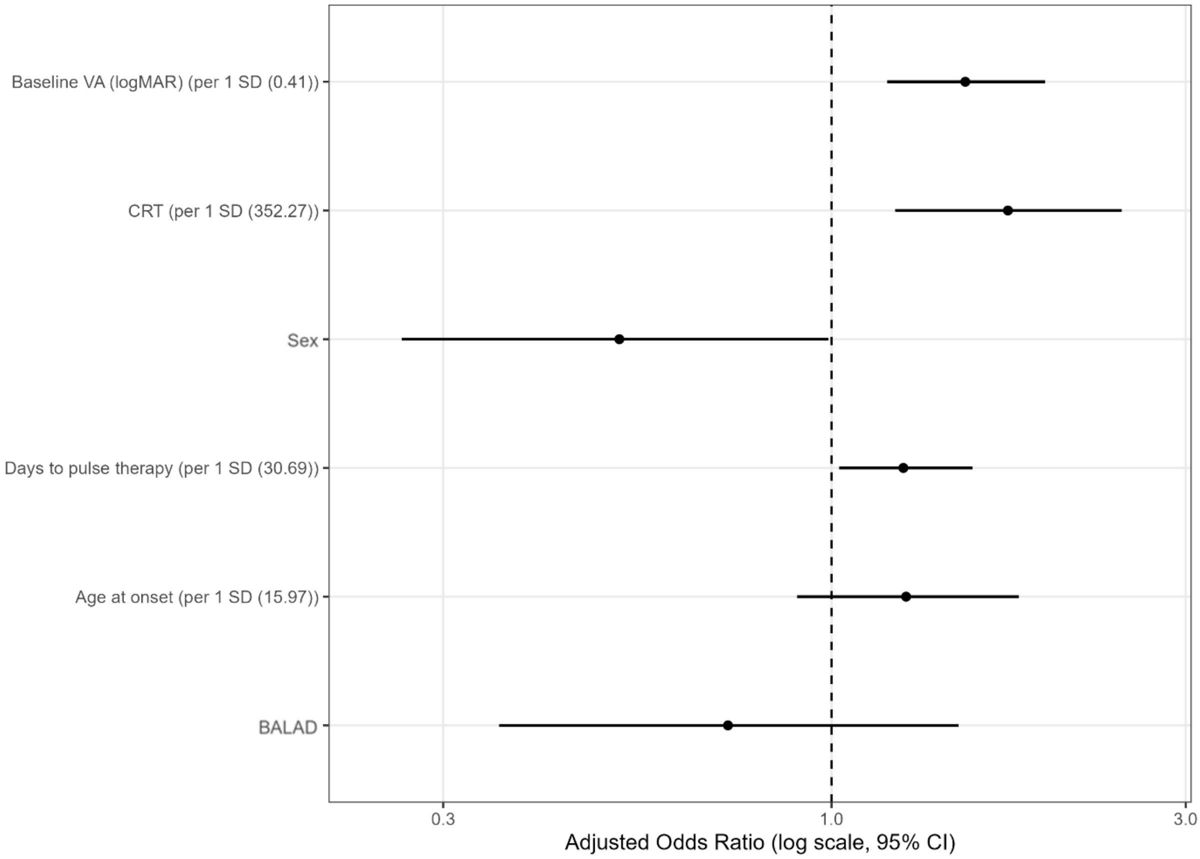

Supplementary Figure S1. Sensitivity analysis including bacillary layer detachment (BALAD) using Firth's penalized logistic regression.

Forest plot of the sensitivity analysis in which bacillary layer detachment (BALAD) was added to the multivariable logistic regression model, applying Firth's penalized method to address potential sparse data bias. Continuous variables were standardized and expressed per 1 standard deviation (SD). Binary variables were analyzed as present vs. absent (or female vs. male for sex). Odds ratios (OR) are presented with 95% confidence intervals (CI). Detailed numerical results are provided in Supplementary Table S1.

Abbreviations: VA = visual acuity; CRT = central retinal thickness; BALAD = bacillary layer detachment.
