# Supplementary material for: Clinical and OCT Predictors of Refractory Vogt–Koyanagi–Harada Disease
Source: Ophthalmol Sci. 2026 Feb 18;6(4):101123. doi: 10.1016/j.xops.2026.101123 (PMC13011244; doi:10.1016/j.xops.2026.101123)
Supplement: Figure S2 [file mmc4.pdf]

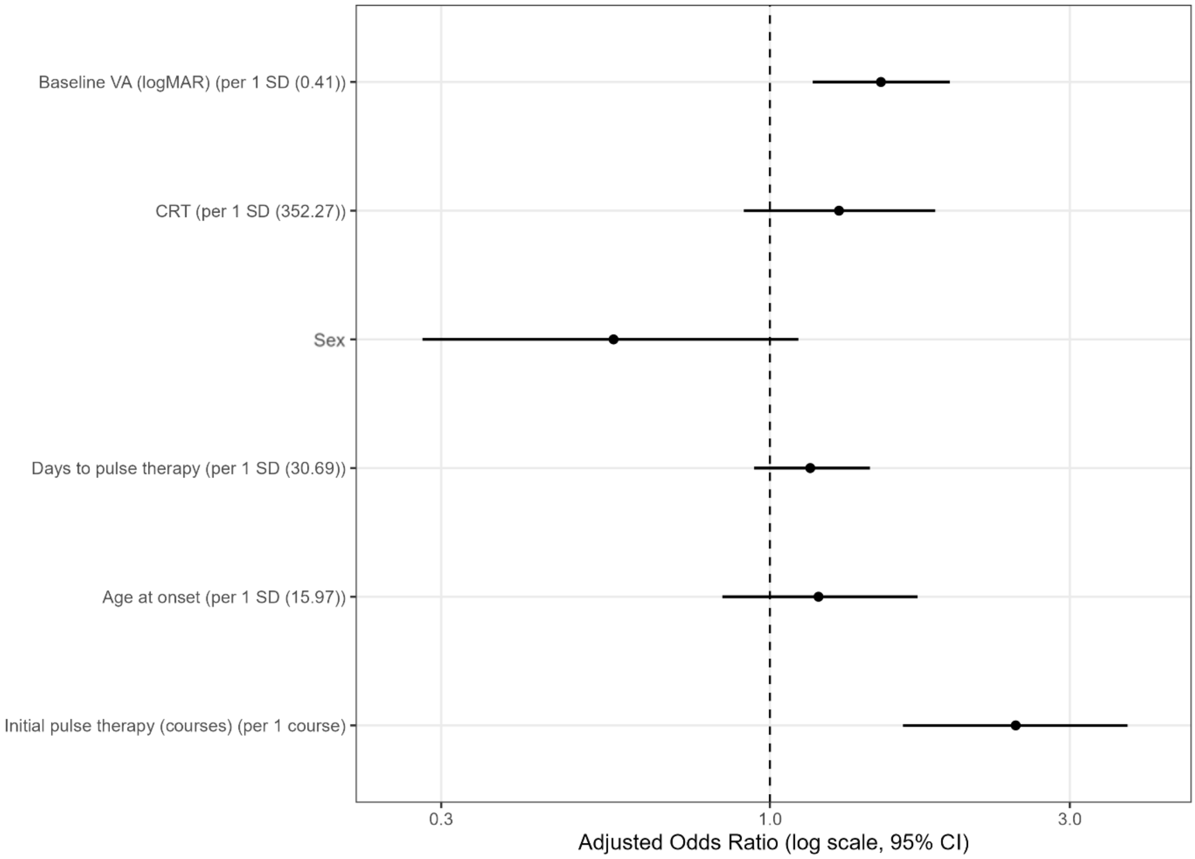

Supplementary Figure S2. Sensitivity analysis including the number of initial pulse therapy courses using Firth's penalized logistic regression.

Forest plot of the sensitivity analysis in which the number of initial pulse therapy courses was added to the multivariable logistic regression model, applying Firth's penalized method to address potential sparse data bias. Continuous variables were standardized and expressed per 1 standard deviation (SD). The number of initial pulse therapy courses was analyzed per additional course. Binary variables were analyzed as present vs. absent (or female vs. male for sex). Odds ratios (OR) are presented with 95% confidence intervals (CI). Detailed numerical results are provided in Supplementary Table S2.

Abbreviations:

VA = visual acuity; CRT = central retinal thickness.
